# Elevated Factor VIII and von Willebrand Factor Levels Predict Unfavorable Outcome in Stroke Patients Treated with Intravenous Thrombolysis

**DOI:** 10.3389/fneur.2017.00721

**Published:** 2018-01-23

**Authors:** Noémi Klára Tóth, Edina Gabriella Székely, Katalin Réka Czuriga-Kovács, Ferenc Sarkady, Orsolya Nagy, Levente István Lánczi, Ervin Berényi, Klára Fekete, István Fekete, László Csiba, Zsuzsa Bagoly

**Affiliations:** ^1^Division of Clinical Laboratory Sciences, Department of Laboratory Medicine, Faculty of Medicine, University of Debrecen, Debrecen, Hungary; ^2^Department of Neurology, Faculty of Medicine, University of Debrecen, Debrecen, Hungary; ^3^Department of Laboratory Medicine, Faculty of Medicine, University of Debrecen, Debrecen, Hungary; ^4^Department of Radiology, Faculty of Medicine, University of Debrecen, Debrecen, Hungary; ^5^MTA-DE Cerebrovascular and Neurodegenerative Research Group, Debrecen, Hungary

**Keywords:** acute ischemic stroke, intracerebral hemorrhage, factor VIII, therapy outcome, thrombolysis, von Willebrand factor

## Abstract

**Introduction:**

Plasma factor VIII (FVIII) and von Willebrand factor (VWF) levels have been associated with the rate and severity of arterial thrombus formation and have been linked to outcomes following thrombolytic therapy in acute myocardial infarction patients. Here, we aimed to investigate FVIII and VWF levels during the course of thrombolysis in acute ischemic stroke (AIS) patients and to find out whether they predict long-term outcomes.

**Materials and methods:**

Study population included 131 consecutive AIS patients (median age: 69 years, 60.3% men) who underwent i.v. thrombolysis with recombinant tissue plasminogen activator (rt-PA). Blood samples were taken on admission, 1 and 24 h after rt-PA administration to measure FVIII activity and VWF antigen levels. Neurological deficit of patients was determined according to the National Institutes of Health Stroke Scale (NIHSS). ASPECT scores were assessed using computer tomography images taken before and 24 h after thrombolysis. Intracranial hemorrhage was classified according to the European Cooperative Acute Stroke Study (ECASS) II criteria. Long-term functional outcome was determined at 90 days after the event by the modified Rankin scale (mRS).

**Results:**

VWF levels on admission were significantly elevated in case of more severe AIS [median and IQR values: NIHSS <6:189.6% (151.9–233.2%); NIHSS 6–16: 199.6% (176.4–250.8%); NIHSS >16: 247.8% (199.9–353.8%), *p* = 0.013]; similar, but non-significant trend was observed for FVIII levels. FVIII and VWF levels correlated well on admission (*r* = 0.748, *p* < 0.001) but no significant correlation was found immediately after thrombolysis (*r* = 0.093, *p* = 0.299), most probably due to plasmin-mediated FVIII degradation. VWF levels at all investigated occasions and FVIII activity before and 24 h after thrombolysis were associated with worse 24 h post-lysis ASPECT scores. In a binary backward logistic regression analysis including age, gender, high-sensitivity C-reactive protein, active smoking, diabetes, and NIHSS >5 on admission, elevated FVIII and VWF levels after thrombolysis were independently associated with poor functional outcomes (mRS ≥ 3) at 90 days (immediately after thrombolysis: FVIII: OR: 7.10, 95% CI: 1.77–28.38, *p* = 0.006, VWF: OR: 6.31, 95% CI: 1.83–21.73, *p* = 0.003; 24 h after thrombolysis: FVIII: OR: 4.67, 95% CI: 1.42–15.38, *p* = 0.011, VWF: OR: 19.02, 95% CI: 1.94–186.99, *p* = 0.012).

**Conclusion:**

Elevated FVIII activity and VWF antigen levels immediately after lysis and at 24 h post-therapy were shown to have independent prognostic values regarding poor functional outcomes at 90 days.

## Introduction

Stroke is the second leading cause of death worldwide and the most common cause of disability (1). Currently, intravenous administration of recombinant tissue plasminogen activator (rt-PA), initiated within 4.5 h after symptom onset, is the standard therapy for the treatment of acute ischemic stroke (AIS). Although thrombolysis using rt-PA treatment has been proven to be safe and effective by a number of clinical trials and systematic reviews (2, 3), only approximately half of treated patients achieve functional independence with complete or nearly complete neurological recovery at 3 months (4). Of all thrombolysis-related complications, the most feared is symptomatic intracerebral hemorrhage, which occurs in approximately 7% of patients causing significant morbidity and mortality (5, 6). Several factors have been associated with a potentially increased risk of poor outcome after rt-PA treatment (e.g., advanced age, male gender, stroke severity, diabetes, baseline hyperglycemia, etc.) (5), still, clinical outcomes are not easily foreseen at the initiation of the therapy as most of the current clinical–radiological risk scores are non-specific and have modest predictive value (6). In order to improve patient care in the near future, new risk prediction models incorporating the use of specific biomarkers might be helpful. A potentially useful biomarker in this case is expected to be involved in the underlying pathophysiological pathway and must confer significant predictive value regarding therapy outcomes.

A growing body of research has demonstrated a role for coagulation factor VIII (FVIII) and von Willebrand factor (VWF) in the pathophysiology of AIS (7, 8). FVIII is stabilized in the circulation by VWF: the linear relationship between the levels of FVIII and VWF has been described (9). The FVIII–VWF complex plays an important role in the propagation of thrombus formation as it mediates platelet attachment to the damaged subendothelium and supports the activation of coagulation cascade at the site of the growing thrombus (7). Plasma FVIII/VWF levels have been directly linked to the rate and severity of arterial thrombus formation (10). Elevated levels of FVIII/VWF have been associated with the risk of stroke (8, 11, 12); however, less evidence is available on the prognostic value of both markers in stroke outcomes (12, 13). Only few papers, including relatively small cohort of patients describe a potential relationship between FVIII and/or VWF levels and thrombolysis outcomes (14). Elevated levels of VWF during thrombolysis in acute myocardial infarction (AMI) patients have been associated with poor recanalization and worse outcomes (15). Less is known about the levels of FVIII and VWF during the course of thrombolysis in AIS patients—even though it has been speculated based on animal studies that the degradation of FVIII during thrombolysis treatment could be a potential causative factor for bleeding complications occurring in treated patients (16).

In the past decades, inhibition of the action of VWF has become an attractive new antithrombotic strategy in ischemic stroke management (7, 17). When used in combination with rt-PA, VWF antagonists could promote the rapid dissolution of the occluding thrombus and limit subsequent thrombo-inflammatory ischemia/reperfusion injury (17). As most of these studies were performed in animal models, data regarding the changes of VWF/FVIII levels during thrombolysis in humans could be of particular interest.

Here, we hypothesized that levels of FVIII and VWF are related to stroke severity and/or patient outcome following thrombolysis treatment. In this observational study, we investigated the levels of FVIII and VWF in a cohort of consecutive AIS patients using blood samples taken on three occasions during the course of i.v. thrombolysis and assessed the potential prognostic value of the measured parameters on functional outcomes at 90 days post-event.

## Materials and Methods

### Patients

Consecutive AIS patients aged 18 years or more, eligible for thrombolysis, admitted to a single Stroke Center (Department of Neurology, Faculty of Medicine, University of Debrecen, Hungary) were enrolled in the study. All patients were within 4.5 h of their symptom onset at the time of admission. Patient enrollment lasted for 22 months starting in March 2011. Intravenous thrombolytic therapy was applied according to the European Stroke Organization (ESO) guidelines using rt-PA (Alteplase, Boehringer Ingelheim, Germany) (18). Inclusion and exclusion criteria of patients were identical to that of thrombolysis eligibility as described in the ESO 2008 guideline (18). The diagnosis of IS was based on clinical symptoms and brain imaging using computer tomography (CT) scan and CT angiography (CTA). Admission CTA was used to identify the level of vessel occlusion in every patient. A control CT was performed for every patient 24 h after thrombolysis. All CT images were analyzed by four different investigators blinded to the clinical state of the patients and the Alberta Stroke Program Early CT Scores (ASPECTS) were calculated (19). Neurological deficit of patients was determined by the NIHSS (20) at various time points: on admission and at 2 h, 24 h, and 7 days post-lysis. Stroke etiology was determined according to the Trial of ORG 10172 in Acute Stroke Treatment (TOAST) criteria (21). Hemorrhagic events were classified as symptomatic or asymptomatic intracranial hemorrhage (SICH or aSICH, respectively) according to the European Cooperative Acute Stroke Study (ECASS) II criteria (22). For each patient a detailed list of clinical parameters was recorded including demographic characteristics, neurological status, time of symptom onset, cardiovascular risk factors (arterial hypertension, atrial fibrillation, hyperlipidemia, diabetes mellitus, smoking status), history of previous cardiovascular events, and medications. Patients were followed and long-term functional outcomes were determined at 90 days post-event using the modified Rankin Scale (mRS) (23).

The following outcomes were investigated: (1) short-term functional outcome at 7 days post-event: favorable outcome was defined as a decrease in NIHSS score by at least 4 points or to 0 by day 7, unfavorable outcome was defined as an increase in NIHSS score by at least 4 points by day 7. (2) The presence of therapy-associated SICH or aSICH was defined according to ECASS II criteria. (3) Long-term functional outcome at 90 days post-event: poor long-term outcome was specified as an mRS greater than 2.

The study was approved by the Ethics Committee of the University of Debrecen, Hungary. All patients or their relatives provided written informed consent.

### Blood Sampling and Laboratory Measurements

Peripheral blood samples were drawn from patients into vacutainer tubes on three different occasions: upon hospital admission (before thrombolysis), immediately after the administration of rt-PA infusion (~1 h after the initiation of thrombolysis) and approximately 24 h after the administration of thrombolytic therapy. Routine laboratory tests were performed from blood samples taken before thrombolysis and included the measurements of complete blood count, serum ions, glucose levels, basic kidney function tests, liver function test, high-sensitivity C-reactive protein (hsCRP), and screening tests of coagulation (prothrombin time, activated partial thromboplastin time and thrombin time). Blood samples anticoagulated with sodium citrate, theophylline, adenosine and dipyridamole (Vacuette CTAD Tubes, Greiner Bio-One, Austria) were centrifuged at 1,220 *g*, room temperature for 15 min. Plasma aliquots were labeled with a code and stored at −70°C until further analysis of FVIII activity and VWF antigen levels. FVIII activity, determined by chromogenic method and VWF antigen level, determined by immunoturbidimetric assay were measured from coded plasma samples on a BCS coagulometer by standard methods (Siemens Healthcare Diagnostic Products, Marburg, Germany), by an investigator blinded to patient identification and clinical information. Batches of plasma samples (approximately 20 samples) were thawed for 10 min at 37°C followed by immediate analysis. According to the manufacturer, precision of the assays are the following: coefficient of variation within run (intra-assay precision) for FVIII activity assay: 4–9%, for VWF antigen level test: 1.4–4.2%; between run (inter-assay precision) for FVIII activity assay: 4–10%, for VWF antigen level test: 0.9–2.6%.

### Statistical Analysis

Statistical analysis was performed using the Statistical Package for Social Sciences (SPSS, Release 22.0, Chicago, IL, USA) and GraphPad Prism 5.0 (GraphPad Prism Inc., La Jolla, CA, USA) softwares. Normality of the data was evaluated by the Shapiro–Wilk test. As FVIII activity and VWF antigen levels were not normally distributed at any time points measured, the Mann–Whitney *U* test was applied for all two-group analyses and the Kruskal–Wallis analysis with Dunn–Bonferroni *post hoc* test was used for multiple comparisons. Differences between categorical variables were assessed by the Fisher’s exact or χ^2^ test. Friedman’s two-way ANOVA with Dunn–Bonferroni *post hoc* test was applied to investigate the effect of thrombolysis on FVIII activity and VWF levels. Strength of association between FVIII activity and VWF antigen levels was tested using Spearman’s correlation test. In order to test for differences between adjusted means, univariate analysis incorporating covariate testing (one-way ANCOVA) was performed after logarithmic transformation of data. Positive predictive values (PPVs) and negative predictive values (NPVs) of the studied parameters were assessed using contingency tables and the Fisher’s exact test. A binary backward logistic regression model was used to determine whether elevated FVIII and VWF levels of different time points are independent predictors of poor functional outcomes at 90 days post-event. Adjustment of the models were based on the results of previous statistical analyses (Mann–Whitney *U* test, Fisher’s exact, or χ^2^ test), previous literature and methodological principles (dichotomized variables wherever possible). Results of the logistic regression analysis were expressed as odds ratio (OR) and 95% confidence interval (CI). A *p*-value of <0.05 was considered statistically significant.

## Results

### Study Population

During the study period, 131 consecutive AIS patients receiving intravenous rt-PA treatment were enrolled. Baseline demographic variables, main stroke characteristics and details of thrombolysis treatment are listed in Tables 1 and 2. In case of six patients, intravenous thrombolytic therapy was supplemented with intra-arterial thrombolysis using rt-PA according to standard protocol; the duration of thrombolysis and the final dose of rt-PA applied did not significantly differ for these patients. Median age of the patient cohort was 69 (IQR: 59–79) years, 60.3% were men. The most common cerebrovascular risk factor was arterial hypertension in this patient cohort (*n* = 100, 76.3%). Median time from symptom onset to treatment was 155 (IQR: 125–180) min. Median NIHSS before stroke treatment was 8 (IQR: 5–14). According to the TOAST criteria, etiology of stroke was most commonly large vessel disease (*n* = 49, 37.4%), followed by 27 (20.6%) patients with cardioembolic stroke. As detected by CTA on admission, 70 patients (53.4%) had a vessel occlusion, and 27 patients (20.6%) stenosis. Poor functional outcome at 7 days post-event was observed in 20 cases (15.3%), while poor outcome at 90 days (mRS ≥ 3) was observed in case of 51 (38.9%) patients. Therapy-associated intracranial hemorrhage was detected in 13 cases, of which 6 cases (4.6%) were symptomatic according to ECASS II. Mortality rates by day 7, 14, and day 90 post-event were 3.8, 6.1, and 22.1%, respectively.

**Table 1:** Study population.

Variable	
Patients, *n*	131
Age, median (IQR)	69.0 (59.0–79.0)
Male, *n* (%)	79 (60.3)
**Cerebrovascular risk factors, *n* (%)**	
Arterial hypertension	100 (76.3)
Atrial fibrillation	35 (26.7)
Hyperlipidemia	81 (61.8)
Diabetes mellitus	39 (29.8)
Smoking	
Non-smoker	69 (52.7)
Previous smoker	16 (12.2)
Current smoker	31 (23.7)
Undetermined	15 (11.5)
**Previous stroke or TIA, *n* (%)**	42 (32.1)
**Thrombolysis treatment, median (IQR)**	
Time from symptom onset to treatment (min)	155 (125.0–180.0)
Duration of thrombolysis (min)	60 (60.0–65.0)
rt-PA dose (mg)	67.0 (58.0–80.8)
**Medication at enrollment, *n* (%)**	
Antihypertensive therapy	93 (71.0)
Angiotensin-converting enzyme inhibitor	60 (45.8)
Alpha blocker	7 (5.3)
Beta blocker	56 (42.8)
Calcium channel blocker	30 (22.9)
Diuretics	39 (29.8)
Antiplatelet drug^a^	58 (44.3)
Anticoagulant drug	7 (5.3)
Lipid lowering therapy	38 (29.0)
Antidiabetic therapy^b^	16 (12.2)
**Laboratory measurements, median (IQR)**	
INR	0.98 (0.94–1.03)
APTT (s)	28.5 (26.1–32.1)
WBC (G/L)	7.59 (6.12–9.0)
Platelets (G/L)	207.5 (169.0–254.3)
Serum glucose (mmol/L)	6.5 (5.5–7.9)
hsCRP (mg/L)	3.06 (1.7–5.9)
Creatinine (μmol/L)	78.0 (64.0–97.0)

*IQR, interquartile range; TIA, transient ischemic attack; rt-PA, recombinant tissue plasminogen activator; INR, international normalized ratio; APTT, activated partial thromboplastin time; WBC, white blood cell; hsCRP, high sensitivity CRP*.

*^a^Aspirin or P2Y12 inhibitor treatment or both*.

*^b^Insulin therapy or oral antidiabetic drug therapy*.

**Table 2:** Stroke characteristics.

Variable	
**Stroke severity on admission, *n* (%)**	
NIHSS 0–5	36 (27.5)
NIHSS 6–10	46 (35.1)
NIHSS 11–16	29 (22.1)
NIHSS >16	17 (13.0)
Undetermined	3 (2.3)
**Stroke etiology (TOAST), *n* (%)**	
Large artery atherosclerosis	49 (37.4)
Small vessel occlusion	13 (9.9)
Cardioembolic	27 (20.6)
Other/undetermined	42 (32.1)
**Imaging data**	
ASPECTS, median (IQR)	
On admission	10 (9–10)
24 h after thrombolysis	9 (5–10)
Affected vessel territory, *n* (%)	
MCA	82 (62.6)
ICA	11 (8.4)
MCA + ICA	10 (7.6)
VB	28 (21.4)
Level of occlusion, *n* (%)	
No stenosis/occlusion	34 (26.0)
Stenosis	27 (20.6)
Occlusion	70 (53.4)
**Outcomes, *n* (%)**	
Functional outcome at 7 days	
Favorable outcome	49 (37.4)
No change	42 (32.1)
Unfavorable outcome	20 (15.3)
Functional outcome at 90 days	
mRS 0–2	57 (43.5)
mRS 3–6	51 (38.9)
Undetermined	23 (17.6)
Intracranial hemorrhage (ECASS II)	
aSICH	7 (5.3)
SICH	6 (4.6)

*n, number of patients; IQR, interquartile range, NIHSS, National Institutes of Health Stroke Scale; TOAST, Trial of ORG 10172 in Acute Stroke Treatment; ASPECTS, Alberta Stroke Program Early Computed Tomography Score; MCA, middle cerebral artery; ICA, internal carotid artery; VB, vertebrobasilar territory; mRS, modified Rankin Scale; ECASS II, European Cooperative Acute Stroke Study II; aSICH, asymptomatic intracerebral hemorrhage; SICH, symptomatic intracerebral hemorrhage*.

### The Effect of Thrombolysis on FVIII Activity and VWF Antigen Levels

**Figure 1:**
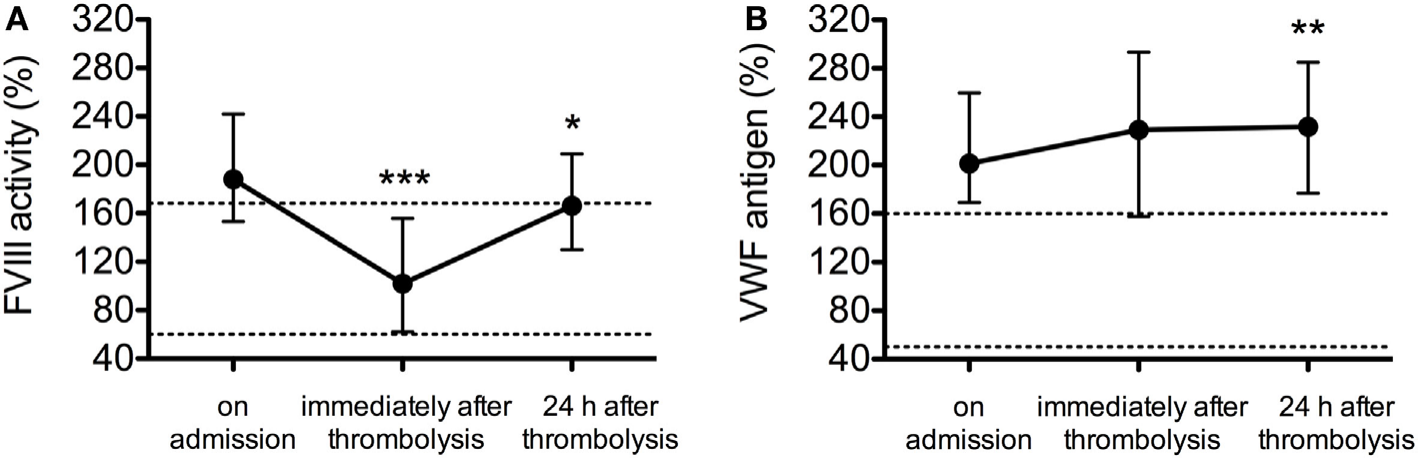
The effect of thrombolytic therapy on factor VIII (FVIII) activity **(A)** and von Willebrand factor (VWF) antigen levels **(B)**. Results are expressed as median values and interquartile ranges (whiskers) as measured from samples taken on admission, immediately after thrombolysis and 24 h after thrombolysis. Upper and lower limits of respective reference intervals are indicated with dashed lines (FVIII activity: 60–168%, VWF antigen levels: 50–160%). Statistical significance was assessed using Friedman’s two-way ANOVA with Dunn–Bonferroni *post hoc* test. *p < 0.05, **p < 0.01, ***p < 0.001 as compared to values on admission.

The median and IQR values of FVIII activity and VWF antigen levels are shown at all investigated time points in Figures 1A,B, respectively. In the samples taken on admission, the median values of both hemostasis parameters were above the upper limit of the respective reference interval in the whole patient cohort (FVIII activity median: 188.0%, IQR: 153.0–242.0%, VWF antigen level median: 201.3%, IQR: 169.1–259.6%). FVIII activity dropped significantly in the samples obtained immediately after thrombolysis as compared to the initial values (median: 102.0%, IQR: 62.0–155.5%, *p* < 0.001) and showed an increase 24 h after the event (median: 166%, IQR: 130.0–209.0%, *p* = 0.014) (Figure 1A). On the contrary, VWF levels increased steadily post-lysis (median VWF levels immediately after lysis: 229.1%, IQR: 157.6–293.3%, at 24 h post-lysis: 231.6%, IQR: 176.8–284.8%, Friedman’s two-way ANOVA *p* = 0.002). Notably, VWF median and IQR values were above the upper limit of the reference interval at all investigated time points in the study cohort (Figure 1B).

**Figure 2:**
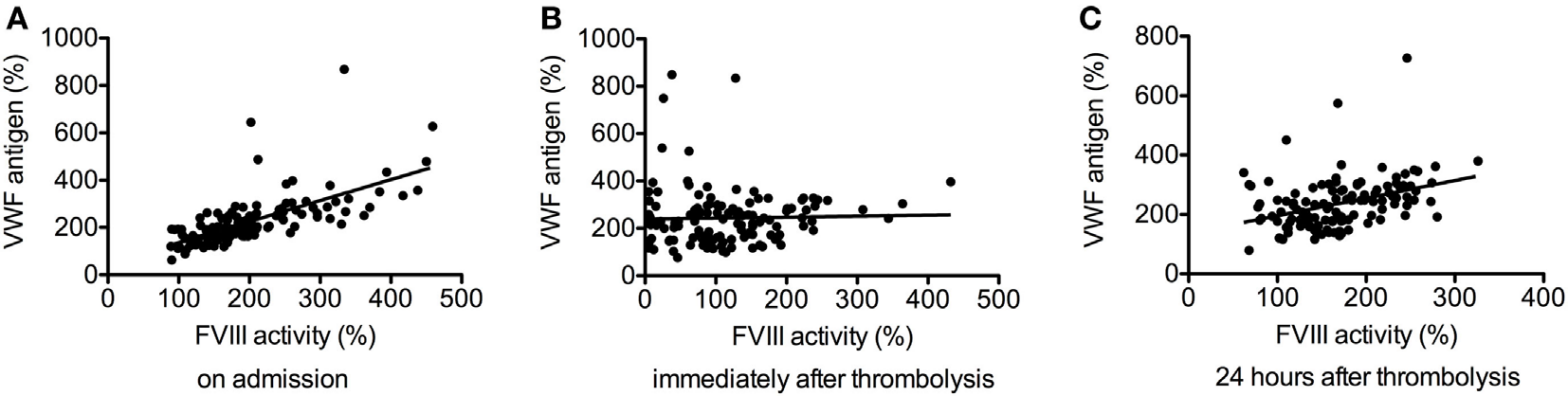
Correlation between factor VIII (FVIII) activity and von Willebrand factor (VWF) antigen levels before thrombolysis (*r* = 0.748, *p* < 0.001) **(A)**, immediately after thrombolysis (*r* = 0.093, *p* = 0.299) **(B)**, and 24 h after thrombolysis (*r* = 0.420, *p* < 0.001) **(C)**.

Factor VIII activity and VWF antigen levels showed good correlation on admission (*r* = 0.748, *p* < 0.001) (Figure 2A), but no significant correlation was found immediately after thrombolysis (*r* = 0.093, *p* = 0.299, Figure 2B), most probably due to plasmin-mediated FVIII degradation. Fair correlation was observed between the two parameters in the samples obtained 24 h after thrombolysis (*r* = 0.420, *p* < 0.001, Figure 2C).

### The Association of FVIII Activity and VWF Antigen Levels with Stroke Severity

von Willebrand factor antigen levels were gradually and significantly elevated in case of more severe AIS (NIHSS 6–16 and NIHSS >16) at all investigated time points, but no such significant association was observed for FVIII activity levels (Table 3). The association between VWF antigen levels and stroke severity remained significant after adjustments for confounders (current smoking, hsCRP, age) in the statistical model.

**Table 3:** FVIII activity and VWF antigen levels according to stroke severity on admission.

Stroke severity on admission					

	NIHSS 0–5 (*n* = 36)	NIHSS 6–16 (*n* = 75)	NIHSS > 16 (*n* = 17)	*p*-Value	*p*-Value*
**FVIII activity (%)**					
On admission	175.5 (145.0–209.3)	186.0 (155.0–238.0)	212.0 (165.5–352.0)	0.162	0.046
Immediately after thrombolysis	108.0 (47.0–148.0)	92.0 (70.0–152.0)	114.0 (51.5–201.0)	0.571	0.400
24 h after thrombolysis	159.0 (115.5–188.5)	162.0 (134.0–208.0)	172.0 (134.0–234.0)	0.292	0.163
**VWF antigen (%)**					
On admission	189.6 (151.9–233.2)	199.6 (176.4–250.8)	247.8 (199.9–353.8)	0.013	0.013
Immediately after thrombolysis	172.2 (141.2–257.0)	229.2 (166.6–293.6)	276.8 (219.1–375.5)	0.008	0.011
24 h after thrombolysis	181.4 (156.9–246.8)	235.9 (188.3–292.7)	261.7 (192.4–366.8)	0.003	0.001

*NIHSS, National Institutes of Health Stroke Scale; FVIII, factor VIII; VWF, von Willebrand factor*.

Data are presented as median and interquartile range. Stroke severity of patients was classified according to previous literature (24, 25). *p*-Value: statistical significance assessed using the Kruskal–Wallis test. *p*-Value*: statistical significance assessed after adjustments for covariates (active smoking, hsCRP, age) in the statistical model (ANCOVA after logarithmic transformation of variables).

The association of elevated VWF levels and more severe AIS was also proved as significantly higher VWF levels were found at all investigated time points in patients presenting with worse 24 h post-lysis CT scans (ASPECTS score 7–0) (Table 4). Similar association was observed for FVIII levels, except for the samples investigated immediately after lysis. Associations for VWF levels and FVIII activity remained significant after adjustments for confounders (current smoking, hsCRP, age) in the statistical model (Table 4). As CT scans on admission are not indicative of stroke severity and have less predictive values as the ASPECTS at 24 h post-lysis, it was not surprising that no significant association was found between the investigated hemostasis parameters and the ASPECTS on admission (Table 4). No association was found between FVIII activity and VWF antigen levels at any time points and stroke subtypes according to TOAST criteria (data not shown). FVIII activity at 24 h post-lysis was significantly elevated in patients with vessel occlusion (median: 175.0%, IQR: 151.5–227.0%) as compared to those with stenosis only (median: 137.0%, IQR: 98.5–175.0%) or without occlusion/stenosis (median: 142.0%, IQR: 115.0–177.0%) (*p* = 0.001) (Table S1 in Supplementary Material), while such association was not observed for VWF levels.

**Table 4:** FVIII activity and VWF antigen levels according to ASPECTS on admission and at 24 h after thrombolysis.

	ASPECTS on admission				ASPECTS 24 h after thrombolysis			
	10 (*n* = 59)	9–0 (*n* = 34)	*p*-Value	*p*-Value*	10–8 (*n* = 55)	7–0 (*n* = 38)	*p*-Value	*p*-Value*
**FVIII activity (%)**								
On admission	171.0 (139.0–206.0)	188.0 (168.5–262.3)	0.072	0.046	171.0 (135.0–192.0)	194.0 (168.0–299.5)	0.002	0.001
Immediately after thrombolysis	90.0 (38.8–147.3)	125.0 (79.0–165.0)	0.056	0.041	92.0 (55.5–147.5)	114.0 (66.0–177.0)	0.141	0.256
24 h after thrombolysis	166.0 (135.0–198.0)	178.0 (134.0–234.0)	0.290	0.171	156.0 (121.0–182.0)	198.0 (158.0–246.0)	0.001	<0.001
**VWF antigen (%)**								
On admission	193.1 (161.6–246.2)	199.4 (173.4–257.1)	0.236	0.182	191.5 (161.6–231.7)	223.2 (175.1–268.6)	0.012	0.018
Immediately after thrombolysis	224.7 (152.9–284.4)	224.5 (171.7–295.9)	0.505	0.270	199.2 (146.3–261.8)	255.2 (200.6–326.3)	0.004	0.002
24 h after thrombolysis	225.5 (180.0–295.9)	242.8 (180.8–270.2)	0.816	0.783	197.8 (174.2–259.6)	257.4 (205.1–320.1)	0.003	0.017

*ASPECTS, Alberta Stroke Program Early Computed Tomography Score; FVIII, factor VIII; VWF, von Willebrand factor*.

Data are presented as median and interquartile range. Patients on admission were dichotomized as having none (ASPECTS = 10) vs. any (ASPECTS = 9–0) early ischemic signs, while patients at 24 h after thrombolysis were dichotomized as having mild/none (ASPECTS 10–8) vs. moderate/severe (ASPECTS 7–0) ischemic signs according to previous literature (26, 27). *p*-Value: statistical significance assessed using the Mann–Whitney *U* test. *p*-Value*: statistical significance assessed after adjustments for covariates (active smoking, hsCRP, age) in the statistical model (ANCOVA after logarithmic transformation of variables).

### Elevated FVIII Activity and VWF Antigen Levels As Predictors of Thrombolysis Outcomes

Factor VIII activity and VWF antigen levels were not associated at any investigated time points with short-term therapy outcomes as assessed by the changes in NIHSS score by day 7 post-lysis (data not shown).

No association was found between FVIII activity or VWF antigen levels at any investigated time points and therapy-associated ICH, except for higher VWF at 24 h post-lysis in patients presenting with SICH (median: 226.8%, IQR: 176.5–279.4% vs. median: 347.5%, IQR: 263.3–372.1% for no bleeding or aSICH vs. SICH, *p* = 0.017) (Figure S1 in Supplementary Material).

Poor functional outcome (mRS ≥3) at 90 days post-event was associated with traditional risk factors including advanced age, increased NIHSS on admission, elevated hsCRP, and the presence of diabetes/diabetes treatment (Table 5). Moreover, as expected, ASPECTS at 24 h post-lysis and the level of vessel occlusion as detected by CTA was also indicative of the long-term outcome. Among the hemostasis parameters investigated at various time points, elevated FVIII activity 24 h post-lysis and elevated VWF antigen level measured immediately after lysis and 24 h after therapy showed significant association with poor outcomes (Table 5). Both parameters, as measured immediately post-lysis and 24 h post-lysis conferred a significant PPV and NPV for poor functional outcomes (highest PPV: VWF 24 after thrombolysis: 0.83; 95% CI: 0.59–0.96, *p* = 0.009 and highest NPV: FVIII immediately after thrombolysis: 0.73; 95% CI: 0.50–0.89, *p* = 0.009).

**Table 5:** Characteristics of patients according to functional outcomes at 90 days following thrombolysis.

Variables	mRS 0–2 (*n* = 57)	mRS 3–6 (*n* = 51)	*p*-Value
Age, median (IQR)	67.0 (58.0–76.0)	76.0 (62.0–83.0)	*0.011*
Male, n (%)	39 (68.4)	27 (52.9)	*0.116*
**Cerebrovascular risk factors, *n* (%)**			
Arterial hypertension	44 (77.2)	39 (76.5)	1.000
Atrial fibrillation	16 (28.1)	14 (27.5)	1.000
Hyperlipidemia	39 (68.4)	26 (51.0)	0.078
Diabetes mellitus	12 (21.1)	20 (39.2)	0.057
Previous stroke	21 (36.8)	14 (28.5)	0.412
Current smoker	12 (21.1)	13 (25.5)	0.817
**Stroke etiology, *n* (%)**			
Small vessel disease	8 (14.0)	3 (5.9)	
Large vessel disease	17 (29.8)	21 (41.2)	0.238
Cardioembolic	13 (22.8)	10 (19.6)	
**NIHSS on admission, median (IQR)**	6 (4–9)	14 (8–19)	<0.001
**Imaging data**			
ASPECTS, median (IQR)			
on admission	10 (9–10)	10 (9–10)	0.482
24 h after thrombolysis	9 (8–10)	7 (2–9)	0.001
Affected vessel territory, *n* (%)			
MCA	33 (57.9)	33 (64.7)	
ICA	4 (7.0)	3 (5.9)	0.093
MCA + ICA	2 (3.5)	7 (13.7)	
VB	18 (31.6)	8 (15.7)	
Level of occlusion, *n* (%)			
No stenosis/occlusion	22 (38.6)	7 (13.7)	
Stenosis	13 (22.8)	9 (17.6)	0.004
Occlusion	22 (38.6)	35 (68.6)	
**Current drug use, *n* (%)**			
Antihypertensive therapy	43 (75.4)	36 (70.6)	0.821
Antiplatelet drug^a^	26 (45.6)	25 (49.0)	0.846
Anticoagulant drug	2 (3.5)	4 (7.8)	0.425
Lipid lowering therapy	17 (29.8)	15 (29.4)	1.000
Antidiabetic therapy^b^	3 (5.3)	10 (19.61)	0.037
**Laboratory measurements, median (IQR)**			
INR	0.97 (0.94–1.02)	0.99 (0.96–1.06)	0.082
APTT (s)	27.9 (25.9–31.2)	28.6 (26.8–32.2)	0.117
WBC (G/L)	7.56 (6.21–8.87)	7.1 (6.06–9.03)	0.545
Platelets (G/L)	209.0 (179.5–240.7)	198.0 (162.0–261.0)	0.562
Serum glucose (mmol/L)	6.5 (5.45–7.40)	6.5 (5.5–8.03)	0.737
hsCRP (mg/L)	2.34 (1.02–4.12)	4.72 (1.80–10.11)	0.002
Creatinine (μmol/L)	77.0 (65.0–90.5)	81.0 (61.0–101.0)	0.735
**FVIII activity (%), median (IQR)**			
On admission	175.0 (142.0–218.5)	191.0 (161.0–274.0)	0.092
Immediately after thrombolysis	88.0 (44.5–149.0)	110.0 (66.0–185.0)	0.102
24 h after thrombolysis	153.0 (120.5–174.0)	176.0 (134.0–237.0)	0.018
**VWF antigen (%), median (IQR)**			
On admission	193.1 (162.1–255.2)	214.0 (176.8–262.2)	0.092
Immediately after thrombolysis	204.1 (141.8–265.8)	254.8 (176.8–323.2)	0.011
24 h after thrombolysis	212.5 (160.0–251.6)	259.2 (191.0–315.1)	0.002

*mRS, modified Rankin Scale; IQR, interquartile range; NIHSS, National Institutes of Health Stroke Scale; ASPECTS, Alberta Stroke Program Early Computed Tomography Score; MCA, middle cerebral artery; ICA, internal carotid artery; VB, vertebrobasilar territory; INR, international normalized ratio; APTT, activated partial thromboplastin time; WBC, white blood cell; hsCRP, high sensitivity CRP; FVIII, factor VIII; VWF, von Willebrand factor*.

*^a^Aspirin or P2Y12 inhibitor treatment or both*.

*^b^Insulin therapy or oral antidiabetic drug therapy*.

A binary backward logistic regression model including age, gender, elevated hsCRP, active smoking, diabetes mellitus, and NIHSS >5 on admission revealed that a FVIII activity and VWF antigen level above the upper limit of the reference interval (168 and 160%, respectively) as measured immediately after lysis and 24 h after thrombolysis significantly and independently increase the risk of unfavorable functional outcomes at 90 days (Table 6). In this model, FVIII activity and VWF antigen levels on admission did not prove to have an independent prognostic value regarding poor functional outcomes at 90 days, while elevated FVIII and VWF levels immediately after thrombolysis conferred an independent OR: 7.09 (IQR: 1.77–28.38, *p* = 0.006) and OR: 6.31 (IQR: 1.83–21.7, *p* = 0.003), respectively. Elevated levels of both factors 24 h after lysis were also found to have a significant predictive value (OR: 4.67, IQR: 1.42–15.38, *p* = 0.011 for FVIII activity and OR: 19.02, IQR: 1.39–187.0, *p* = 0.012 for VWF antigen level). Besides these hemostasis parameters, only hsCRP >5.2 mg/L and NIHSS >5 on admission remained in the stepwise backwards regression analysis model as independent risk factors for poor outcomes at 90 days (OR: 4.85, 95% CI: 1.64–14.33, *p* = 0.004 and OR: 3.51, 95% CI: 1.17–10.57, *p* = 0.026, respectively).

**Table 6:** Association of FVIII activity and VWF antigen levels with poor functional outcome (mRS ≥ 3) at 90 days.

	Odds ratio	95% confidence interval	*p*-Value
**FVIII activity >168%^a^**			
On admission	2.24	0.81–6.21	0.122
Immediately after thrombolysis	7.10	1.77–28.38	0.006
24 h after thrombolysis	4.67	1.42–15.38	0.011
**VWF antigen >160%^a^**			
On admission	2.52	0.65–9.73	0.180
Immediately after thrombolysis	6.31	1.83–21.73	0.003
24 h after thrombolysis	19.02	1.94–186.99	0.012

*mRS, modified Rankin Scale*.

*Backward multiple logistic regression model included age >75 years, gender, hsCRP >5.2 mg/L, active smoking, diabetes mellitus, NIHSS >5 on admission*.

*^a^The upper limit of reference interval*.

## Discussion

In this study, we examined the levels of FVIII and VWF during thrombolysis in 131 consecutive AIS patients and studied the relationship between the hemostasis factor levels and stroke characteristics and therapy outcomes. Only few papers are found in the literature studying the changes of certain hemostasis factors during the course of thrombolysis following ischemic stroke (28–31) and up to our knowledge, none of them studied the levels of FVIII and VWF comprehensively in this respect. It has been known for almost 40 years that *in vitro* plasmin degrades and inactivates FVIII (32). Studies in animal models also suggested such effect of plasmin on FVIII (33); however, the *in vivo* effect of plasmin on FVIII in humans during the course of rt-PA-induced thrombolysis, has not yet been characterized. Here, we showed that FVIII activity drops significantly immediately after thrombolysis as compared to levels measured on admission of patients. However, as the vast majority of patients had elevated FVIII levels on admission, this reduction, most probably due to plasmin-mediated degradation, did not reach a level that would suggest a potential risk for intracerebral hemorrhage. In fact, FVIII levels measured at any time points in this study were not associated with bleeding complications, which is in line with the results of studies in animal models (16). Opposite to FVIII activity, VWF antigen levels showed a rising tendency during the course of thrombolysis in our study. This, in theory might be due to two reasons. The first apparent reason is VWF degradation by plasmin, which has been shown before *in vitro* (34, 35). As the test we used for measuring VWF antigen levels contains polyclonal antibody against VWF, the degradation of the protein leads to an increased antigen level. In an early paper describing the time course of certain hemostasis factors in a few patients (*n* = 7) with AMI treated by rt-PA induced thrombolysis, it was shown that thrombolysis treatment resulted in the elevation of VWF antigen levels, most probably due to the proteolysis of VWF multimers. The degradation of VWF multimers has been speculated to be a potential causative factor for hemorrhagic complications in AMI patients treated with thrombolysis (11, 35). In our study, VWF antigen levels were found to be significantly higher at 24 h post-lysis in patients with SICH as compared to the rest of the cohort, but due to the relatively low number of patients with SICH in this population (*n* = 6), this association should be confirmed by other studies. As for a second reason for the elevation of VWF antigen levels post-lysis, it is plausible that the increase is due to endothelial damage caused by ischemic damage. Studies in AMI patients suggested that thrombolysis induced by streptokinase is associated with an increase of VWF antigen levels due to endothelial damage as a result of oxidative stress caused by the thrombolytic agent (11, 15). Interestingly, in our study, VWF antigen levels showed an increase after thrombolysis only in patients with more severe stroke (NIHSS 6–16 and NIHSS >16 on admission), while in the group of patients with less severe stroke (NIHSS 0–5) this elevation was not seen, suggesting that at least in part endothelial dysfunction is likely to contribute to this finding.

Many studies have investigated the association between FVIII and/or VWF levels and the risk of cardiovascular or cerebrovascular events (8, 9, 12, 36). Despite few conflicting results, it has been well established that elevated FVIII and/or VWF levels predispose patients to AIS (8, 12). In line with our findings, most studies revealed that in the majority of tested patients with AIS, high FVIII and VWF levels were found; moreover, baseline stroke severity, as measured by the NIHSS score was associated with elevated FVIII and/or VWF levels (13, 37, 38). Furthermore, beyond these previously known results, here we describe a strong association between elevated FVIII/VWF levels during the course of thrombolysis and the ASPECT score in patients as assessed 24 h post-lysis. The only non-significant association in this respect was for FVIII activity tested immediately after lysis, which was most probably due to plasmin-mediated degradation of the protein. The finding that the ASPECT score the day after stroke shows a strong association with the tested hemostasis parameters is of considerable interest, as it indicates a link between the investigated factors and stroke severity as verified by not only the NIHSS functional score but by imaging analysis as well. Similar findings on the relation of any hemostasis factors and the results of such imaging analysis is practically lacking in the literature as yet. Limited evidence is available on the possible association of FVIII/VWF levels with the etiology of stroke; moreover, reports are often discordant in this respect (8, 12, 39). Here, we could not find any association of FVIII/VWF levels with the subtype of stroke as classified by the TOAST criteria.

While the association between VWF levels and thrombolysis outcome following AMI has been studied before (15), surprisingly, similar data regarding ischemic stroke are much more limited. Here, we show that a FVIII activity and VWF antigen level above the upper limit of the reference interval, as measured immediately after or 24 h post-lysis, confer a significant, independent risk for poor functional outcomes at 90 days post-event. Our results indicate that both factors could be useful biomarkers having significant prognostic values on long-term outcomes, which might help with patient selection requiring alternative treatment post-lysis. At the same time, at least according to results of this cohort, pretreatment FVIII and VWF levels were not indicative of thrombolysis outcomes.

Although here we propose that our results have prognostic value in the studied patient cohort; nevertheless, we consider the relevance of our findings as potentially useful descriptive data, which might provide basis for future research, while its clinical relevance remains to be fully elucidated. In the era of mechanical thrombectomy, the management of AIS faces new types of decision-making questions in the clinical practice. Useful biomarkers with predictive values regarding poor outcomes might be incorporated into algorithms designed to select candidates for alternative approaches rather than rt-PA alone, e.g., mechanical thrombectomy or other pharmacological approaches (40–42). Moreover, studies on VWF in particular might prove even more useful in the future, as preclinical and clinical studies on inhibitors of VWF are promising and show a safe antithrombotic potential. When used in combination with tPA, VWF antagonists were able to prevent ongoing microvascular thrombus formation reducing stroke progression (7, 17). These findings are particularly interesting in the light of our observations showing that VWF median and IQR values were constantly above the upper limit of the reference interval in the studied AIS population. Although drugs that target the inhibition of the action of VWF have not yet reached approval for the market (17), studies on FVIII/VWF levels during stroke and thrombolysis might provide useful descriptive data to understand the pathophysiology relevant to the potential clinical application of these inhibitors in the future in humans.

### Limitations

Our study has limitations. The sample size is limited, but in the light of other studies on AIS patients treated by thrombolysis it is considered representative. Due to the limited number of patients with SICH and with poor outcomes in this cohort, despite the significant associations found, results presented here need to be verified by larger studies.

We did not investigate AIS patients who were not suitable for rt-PA therapy. In theory, measuring FVIII/VWF levels of patients receiving and not receiving rt-PA and comparing the results with outcomes might seem useful. However, due to the important baseline differences between the two groups (e.g., the group not receiving rt-PA might be highly heterogeneous regarding time window from symptom onset, baseline coagulation screening tests, effective anticoagulation, age, etc.), which may significantly affect the results, such comparison might fail to support relevant conclusions.

The lack of advanced neuro-imaging (e.g., perfusion and collateral circulation imaging) limits the application of this study.

Finally, as the study was designed to find potential biomarkers with predictive values for long-term outcomes following thrombolysis, we did not perform any functional characterization studies on the plasmin-mediated effect of FVIII and VWF proteins. In case of FVIII activity, we assumed, based on previous studies, that the reduction in activity levels as detected immediately after thrombolysis is due to plasmin-mediated degradation and did not perform any biochemical tests to prove this hypothesis. In case of VWF, functional activity tests, including ristocetin-induced activity tests, collagen-binding assay, multimer testing, etc. were not measured in the patient population. Future studies are required to elucidate whether the qualitative changes of both proteins following stroke and thrombolysis would have any pathophysiological relevance and prognostic value.

## Conclusion

Here, we report the changes in FVIII activity levels and VWF antigen levels during the course of thrombolysis in a cohort of consecutive AIS patients. Elevated FVIII activity and VWF antigen levels immediately after lysis and 24 h post-therapy were shown to have independent prognostic values regarding poor functional outcomes at 90 days.

## Ethics Statement

The study was approved by the Ethics Committee of the University of Debrecen, Hungary. All patients or their relatives provided written informed consent.

## Author Contributions

LC and ZB designed the study. ES, KC-K, FS, EB, KF, and IF were involved in sample collection and source data preparation. NT and ON performed the measurements. NT and ZB analyzed the data, designed and performed the statistical analysis, and wrote the paper. All authors agreed to the final version of the manuscript.

## Conflict of Interest Statement

The authors declare that the research was conducted in the absence of any commercial or financial relationships that could be construed as a potential conflict of interest.

## Supplementary Material

Figure S1 von Willebrand factor (VWF) antigen levels during the course of thrombolysis in patients with or without therapy-associated symptomatic intracranial hemorrhage (SICH). Results are expressed as median values (circles) and interquartile ranges (whiskers) as measured from samples taken on admission, immediately after thrombolysis and 24 h after thrombolysis. Open circles represent patients with SICH, solid circles represent patients with no bleeding or with asymptomatic intracranial hemorrhage (aSICH). Upper and lower limits of VWF reference interval (50–160%) are indicated with dashed lines. Statistical significance was assessed using Mann–Whitney’s *U* test. **p* < 0.05 for SICH vs. no bleeding/aSICH.
