# Supplementary figures and images for: Elevated Factor VIII and von Willebrand Factor Levels Predict Unfavorable Outcome in Stroke Patients Treated with Intravenous Thrombolysis
Source: Front Neurol. 2018 Jan 23;8:721. doi: 10.3389/fneur.2017.00721 (PMC5787073; doi:10.3389/fneur.2017.00721)

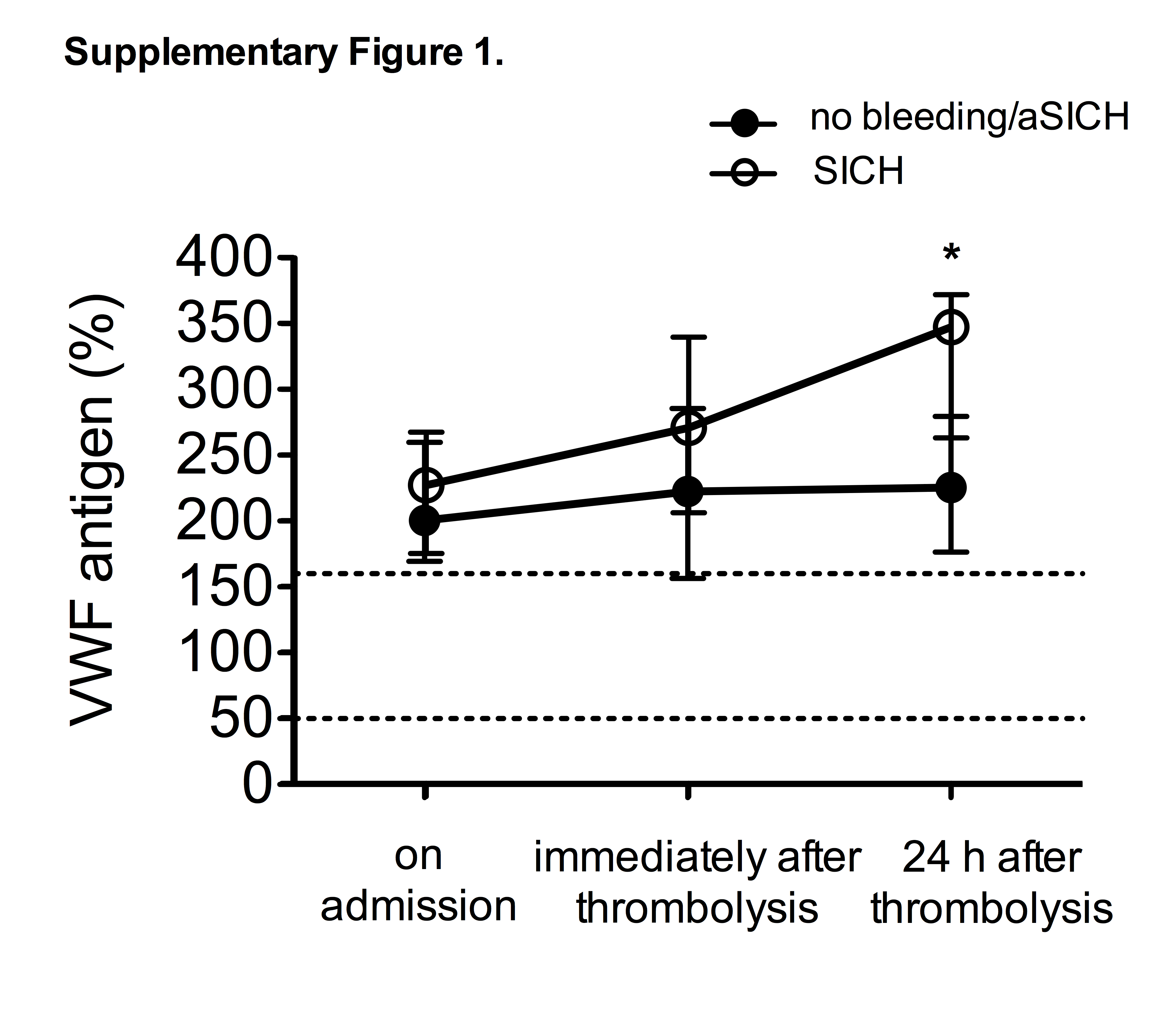

Supplement: Figure S1 — von Willebrand factor (VWF) antigen levels during the course of thrombolysis in patients with or without therapy-associated symptomatic intracranial hemorrhage (SICH). Results are expressed as median values (circles) and interquartile ranges (whiskers) as measured from samples taken on admission, immediately after thrombolysis and 24 h after thrombolysis. Open circles represent patients with SICH, solid circles represent patients with no bleeding or with asymptomatic intracranial hemorrhage (aSICH). Upper and lower limits of VWF reference interval (50–160%) are indicated with dashed lines. Statistical significance was assessed using Mann–Whitney’s U test. *p < 0.05 for SICH vs. no bleeding/aSICH. [file Image_1.tiff]
