# Supplementary material for: Elevated Factor VIII and von Willebrand Factor Levels Predict Unfavorable Outcome in Stroke Patients Treated with Intravenous Thrombolysis
Source: Front Neurol. 2018 Jan 23;8:721. doi: 10.3389/fneur.2017.00721 (PMC5787073; doi:10.3389/fneur.2017.00721)
Supplement: Supplementary file 2 [file Table_1.PDF]

**Supplementary Table 1.** Characteristics of patients according to the level of occlusion as assessed by CT angiography on admission.

| **Variables** | **no stenosis/ occlusion (n=34)** | **stenosis (n=27)** | **occlusion (n=70)** | **P-value** |
|---|---|---|---|---|
| Age, median (IQR) | 67.0 (59.0-75.0) | 70.0 (66.0-82.0) | 70.5 (58.0-80.3) | 0.249 |
| Male, n (%) | 21 (61.8) | 18 (66.7) | 40 (57.1) | 0.680 |
| **Cerebrovascular risk factors, n (%)** | | | | |
| Arterial hypertension | 26 (76.5) | 20 (74.1) | 54 (77.1) | 0.962 |
| Atrial fibrillation | 10 (29.4) | 7 (25.9) | 18 (25.7) | 0.964 |
| Hyperlipidaemia | 24 (70.6) | 20 (74.1) | 37 (52.9) | 0.078 |
| Diabetes mellitus | 12 (35.3) | 8 (29.6) | 19 (27.1) | 0.714 |
| Previous stroke | 12 (35.3) | 13 (48.1) | 17 (24.3) | 0.094 |
| Current smoker | 7 (20.6) | 1 (3.8) | 23 (32.9) | 0.001 |
| **NIHSS on admission, median (IQR)** | 6 (4-8) | 7 (5-9) | 11 (8-16) | <0.001 |
| **Imaging data** | | | | |
| ASPECTS, median (IQR) | | | | |
| on admission | 10 (9-10) | 10 (10-10) | 10 (9-10) | 0.270 |
| 24 h after thrombolysis | 9 (8-10) | 10 (7-10) | 7 (3-9) | 0.003 |
| **Laboratory measurements, median (IQR)** | | | | |
| hsCRP (mg/L) | 2.64 (1.15-4.41) | 3.33 (1.70-5.02) | 3.74 (1.70-8.87) | 0.235 |
| FVIII activity (%) | | | | |
| On admission | 170.0 (143.0-200.0) | 194.0 (168.0-264.0) | 191.0 (155.0-248.0) | 0.138 |
| Immediately after thrombolysis | 99.0 (59.5-141.8) | 89.0 (50.3-150.5) | 110.0 (64.0-178.0) | 0.303 |
| 24 h after thrombolysis | 142.0 (115.0-177.0) | 137.0 (98.5-175.0) | 175.0 (151.5-227.0) | 0.001 |
| VWF antigen (%) | | | | |
| On admission | 192.8 (165.0-223.1) | 205.6 (186.6-285.8) | 199.4 (171.0-260.9) | 0.252 |
| Immediately after thrombolysis | 191.0 (155.3-265.8) | 256.8 (178.8-327.7) | 229.2 (156.4-293.6) | 0.330 |
| 24 h after thrombolysis | 190.4 (166.7-281.3) | 244.5 (170.5-299.3) | 236.0 (189.0-279.2) | 0.297 |

n, number of patients; IQR, interquartile range, NIHSS, National Institutes of Health Stroke Scale; ASPECTS, Alberta Stroke Program Early Computed Tomography Score; hsCRP: high sensitivity C-reactive protein; FVIII: factor VIII; VWF: von Willebrand factor
